# CX3CR1-Dependent Macrophages Drive Ovarian Cancer Progression Through MMP-2 and TGF-β Production

**DOI:** 10.3390/cancers18111855

**Published:** 2026-06-05

**Authors:** Yuko Tanizaki-Horiuchi, Yuko Ishida, Aya Kobayashi, Tamaki Yahata, Yumi Kuninaka, Saori Toujima, Mizuho Nosaka, Reiko Matsuki, Akihiko Kimura, Mika Mizoguchi, Naofumi Mukaida, Kazuhiko Ino, Toshikazu Kondo

**Affiliations:** 1 Department of Forensic Medicine, Wakayama Medical University, Wakayama 641-8509, Japan; yuko-t@wakayama-med.ac.jp (Y.T.-H.); iyuko@wakayama-med.ac.jp (Y.I.); kuninaka@wakayama-med.ac.jp (Y.K.); menosaka@wakayama-med.ac.jp (M.N.); m2588022@wakayama-med.ac.jp (R.M.); legkim@wakayama-med.ac.jp (A.K.); mukaida@wakayama-med.ac.jp (N.M.); 2 Department of Obstetrics and Gynecology, Wakayama Medical University, Wakayama 641-8509, Japan; aya.smile.on.me@gmail.com (A.K.); t-yahata@wakayama-med.ac.jp (T.Y.); toujima@wakayama-med.ac.jp (S.T.); ma-mika@wakayama-med.ac.jp (M.M.); kazuino@wakayama-med.ac.jp (K.I.)

**Keywords:** CX3CL1–CX3CR1, ovarian cancer, peritoneal dissemination, tumor microenvironment, tumor-associated macrophages

## Abstract

**Background:** Epithelial ovarian cancer (EOC) is characterized by aggressive peritoneal dissemination and an immunosuppressive tumor microenvironment in which tumor-associated macrophages (TAMs) play a central role. Chemokine signaling pathways regulate macrophage recruitment and function; however, the contribution of the CX3CL1–CX3CR1 axis to ovarian cancer progression and TAM-mediated effector mechanisms remains unclarified. This study aimed to clarify the role of CX3CL1–CX3CR1 signaling in ovarian cancer progression, focusing on macrophage-derived pro-tumorigenic factors. **Methods:** CX3CL1 and CX3CR1 expression was examined in human EOC and healthy ovarian tissues by real-time polymerase chain reaction and immunohistochemistry. Functional effects of CX3CL1 on ovarian cancer cells were evaluated via migration and proliferation assays in the murine ID8 cell line. An intraperitoneal syngeneic ovarian cancer model was established by injecting ID8 cells into wild-type and Cx3cr1-deficient mice. Tumor burden, ascites formation, survival, macrophage infiltration, and expression levels of matrix metalloprotease-2 (MMP-2) and transforming growth factor-β (TGF-β) were assessed by histological, immunohistochemical, and molecular analyses. **Results:** CX3CL1 and CX3CR1 expression was significantly upregulated in human EOC tissues and associated with marked macrophage infiltration. CX3CL1 stimulation enhanced migration, but not proliferation, of ID8 cells. Cx3cr1 deficiency significantly suppressed intraperitoneal tumor growth, reduced ascitic fluid volume, and prolonged survival. This was accompanied by reduced CX3CR1^+^ TAM accumulation and decreased MMP-2 and TGF-β expression, which were predominantly produced by infiltrating macrophages. **Conclusions:** The CX3CL1–CX3CR1 axis promotes ovarian cancer progression by recruiting MMP-2- and TGF-β-producing macrophages. Targeting CX3CR1-dependent TAM functions may represent a therapeutic strategy for limiting peritoneal dissemination in ovarian cancer.

## Simple Summary

Ovarian cancer is a highly lethal disease because it often spreads widely within the abdominal cavity and is supported by surrounding non-cancerous cells. This study aimed to clarify how a specific signaling pathway, the CX3CL1–CX3CR1 axis, contributes to ovarian cancer progression. Using human tumor samples, mouse models, and cell experiments, we found that this pathway attracted a particular group of macrophages to the tumor environment. These macrophages produce molecules that promote cancer cell invasion and tissue remodeling, thereby accelerating tumor growth and dissemination. Importantly, blocking this pathway reduced tumor burden and abdominal fluid accumulation, and improved survival in mice. Our findings suggest that targeting macrophages recruited by the CX3CL1–CX3CR1 axis, rather than cancer cells alone, may represent a promising new therapeutic strategy for ovarian cancer and could influence future research on tumor–immune cell interactions.

## 1. Introduction

Epithelial ovarian cancer (EOC) is the most fatal gynecological malignancy, accounting for >200,000 deaths annually worldwide [1]. Despite advances in cytoreductive surgery and platinum/taxane-based chemotherapy, most patients experience relapse and chemoresistance, resulting in a poor overall survival [1,2]. Targeted therapies, such as vascular endothelial growth factor (VEGF) and poly (ADP-ribose) polymerase inhibitors, have modestly prolonged progression-free survival; however, durable responses remain limited [2]. Immune checkpoint inhibitors, including anti-PD-1/PD-L1 antibodies, have also shown modest activity against recurrent ovarian cancer [3]. These clinical challenges highlight the urgent need to elucidate the molecular mechanisms driving ovarian cancer progression and identify novel therapeutic targets [4]. Recently, the phase III ENGOT-ov65/KEYNOTE-B96 trial conducted by Colombo et al. [5] demonstrated that pembrolizumab combined with weekly paclitaxel significantly improved progression-free and overall survival in platinum-resistant recurrent ovarian cancer, representing the first evidence of an overall survival benefit with an immune checkpoint inhibitor–based regimen in this disease setting. Nevertheless, the molecular determinants underlying therapeutic response and disease progression remain incompletely understood.

The tumor microenvironment (TME) is now recognized as a pivotal determinant of cancer biology. It is composed of stromal fibroblasts, endothelial cells, extracellular matrix (ECM), and various immune cells [6,7]. Tumor-associated macrophages (TAMs) are the most abundant infiltrating leukocytes in ovarian cancer tissues and ascites [8]. TAMs often acquire an immunosuppressive, pro-tumorigenic phenotype, thereby promoting angiogenesis, ECM remodeling, invasion, and metastasis [5,9,10]. In ovarian cancer, a high M2/M1 macrophage ratio is closely associated with advanced-stage disease, chemoresistance, and poor prognosis [11,12]. Recent studies have emphasized that the functional heterogeneity of TAMs, beyond the classical M1/M2 dichotomy, plays a critical role in shaping tumor progression [13,14,15].

Chemokines orchestrate leukocyte recruitment and polarization in the TME [16]. CX3CL1 (fractalkine) is a unique chemokine that exists in both membrane-bound and soluble forms and signals through the CX3CR1 receptor [17]. CX3CR1 is expressed on multiple immune cell subsets and mediates cell adhesion and migration [17]. In ovarian cancer, CX3CR1 expression in carcinoma cells promotes their motility and adhesion to mesothelial cells, thereby contributing to peritoneal dissemination [10]. High CX3CR1 expression correlates with poor prognosis and increased metastatic potential [18]. Moreover, the hypoxia-induced upregulation of CX3CR1 enhances the invasiveness of ovarian cancer cells [12]. Although CX3CL1 is present in tumor cells and malignant ascites [19], its role in regulating macrophage recruitment and polarization within the ovarian TME remains unclarified [5]. Although CX3CR1 expression has been reported in ovarian cancer cells, the functional role of the CX3CL1–CX3CR1 axis in shaping the tumor microenvironment remains insufficiently characterized. In particular, it is unclear how this signaling pathway regulates the recruitment and functional polarization of tumor-associated macrophages and whether these cells contribute to tumor progression through the production of specific effector molecules such as MMP-2 and TGF-β.

Evidence from other malignancies suggests that CX3CL1–CX3CR1 signaling regulates TAMs. For example, in skin carcinogenesis, this axis recruits M2-like macrophages and promotes tumor growth [20]. Similarly, CX3CR1-expressing macrophages contribute to tumor invasion and metastasis in breast cancer and glioma [21,22]. These findings increase the possibility that CX3CL1–CX3CR1 signaling facilitates ovarian cancer progression by orchestrating the accumulation of macrophages producing tumor-promoting mediators such as matrix metalloprotease-2 (MMP-2) and transforming growth factor-β (TGF-β), which are known to enhance invasion, angiogenesis, and peritoneal dissemination [23,24].

Therefore, we hypothesized that the CX3CL1–CX3CR1 axis plays a central role in ovarian cancer progression by modulating both tumor cell behavior and the macrophage compartment. In this study, we investigated CX3CL1 and CX3CR1 expression in human EOC tissues, examined their functional roles in a syngeneic murine model, and assessed the effects of CX3CR1 deficiency on TAM recruitment and effector functions. Our findings highlight the mechanisms by which this chemokine axis drives peritoneal dissemination and its potential as a therapeutic target.

## 2. Materials and Methods

### 2.1. Reagents and Antibodies

Recombinant murine CX3CL1 chemokine domain was obtained from R&D Systems (Minneapolis, MN, USA). For immunohistochemistry and double immunofluorescence analyses, commercially available antibodies against CX3CR1 (PAB16479, Abnova, Taipei City, Taiwan), F4/80 (T-2006, BMA Biomedicals, Augst, Switzerland), MMP-2 (sc-8835, SantaCruz, Dallas, TX, USA), MMP-9 (sc-6840, SantaCruz), CD8 (550281, clone 53-6.7 (RUO), BD Bioscience, Franklin Lakes, NJ, USA), TGF-β1 (sc-31609, SantaCruz), and VEGF (sc-1836, SantaCruz) were used. Appropriate fluorophore- or enzyme-conjugated secondary antibodies were applied according to the manufacturer’s instructions. For flow cytometric analysis, a PE-conjugated anti-mouse CX3CR1 antibody (FAB5825P, R&D systems) and PE-conjugated anti-mouse F4/80 antibody (clone BM8, 12-4801-82, Thermo Fisher Scientific, Waltham, MA, USA) were used.

### 2.2. Cell Culture

The murine ovarian cancer cell line ID8, originally generated from C57BL/6 mouse ovarian surface epithelium, was kindly provided by Dr. Kathy Roby (University of Kansas Medical Center, Kansas City, KS, USA) [21]. Cells were cultured in MEM-α supplemented with 10% fetal calf serum and antibiotics under standard conditions at 37 °C in a humidified atmosphere with 5% CO_2_.

### 2.3. Cell Proliferation Assay

Cells (4 × 10^3^ cells/well) were cultured in 96-well microplates for 24 and 48 h with or without recombinant CX3CL1. Cell viability was assayed using Cell Counting Kit-8 (CK04, Dojindo Laboratories, Kumamoto, Japan).

### 2.4. Cell Migration Assay

Cell motility was assessed using transwell chambers with 8.0-μm pore size membranes. ID8 cells were seeded into the upper compartments in serum-free medium. The lower chambers contained complete medium with or without recombinant CX3CL1. After incubation, migrated cells on the underside of the membrane were fixed, stained, and quantified microscopically.

### 2.5. Human Ovarian Tissue Samples

Real-time RT-PCR was performed to evaluate the expression of *CX3CL1* and *CX3CR1* in human ovarian tissue samples. Epithelial ovarian cancer (EOC) specimens were obtained from 15 patients who underwent primary surgical treatment for invasive ovarian carcinoma at Wakayama Medical University Hospital as part of routine diagnostic and therapeutic procedures. None of the patients had received neoadjuvant chemotherapy prior to surgery. Histopathological subtypes were classified in accordance with the World Health Organization criteria. Moreover, ovarian tissue samples were also taken from 4 patients with benign serous tumors (range: 40–61 years, median: 48 years) and from 5 patients with non-ovarian diseases (range: 27–64 years, median: 50.5 years). In this experiment, written informed consent was obtained from each patient. All experiments were approved by the Research Ethics Committee of Wakayama Medical University (No. 1509 on 27 October 2014) regarding the use of human samples. All the procedures were performed in accordance with institutional ethical guidelines and the Declaration of Helsinki Principles.

### 2.6. Animals

Female C57BL/6 wild-type mice and *Cx3cr1*-deficient mice (generously gifted by Drs. P. M. Murphy and J. L. Gao (National Institute of Allergy and Infectious Diseases, National Institutes of Health, Bethesda, MD, USA) [25]) on a C57BL/6 background were maintained under specific pathogen-free conditions. All animal experiments were conducted in accordance with institutional guidelines and were approved by the Animal Care and Use Committee of Wakayama Medical University (No. 602 on 1 April 2013).

### 2.7. In Vivo Tumor Generation

Sub-confluent ID8 cells were trypsinized, washed twice, and harvested by centrifugation at 300× *g* for 5 min. A total volume of 1.0 mL containing 5 × 10^6^ cells/mouse was inoculated intraperitoneally into *Cx3cr1^−/−^* and WT mice. The mice were monitored for survival and sacrificed using isoflurane hyperanesthesia on days 79 or 84 after inoculation to assess tumor progression. Before the sacrifice, the mice lived in a manner similar to normal mice. After the sacrifice, laparotomy revealed a small amount of ascites and intraperitoneal tumors, which were collected. The ascites were then centrifuged at 2000× *g* for 5 min to separate the ascites from the ascites cells. The mice were randomly assigned to each experimental group. For downstream analyses, tumor tissues were collected from representative intraperitoneal nodules, primarily from the mesentery and peritoneal surfaces. Tissue sampling was performed in a consistent manner across all experimental groups to ensure comparability. In this study, no in vitro experiments using isolated or differentiated macrophages were performed. The characterization of CX3CR1-dependent macrophages was based on in vivo analyses, including immunohistochemical and immunofluorescence evaluation of tumor tissues.

### 2.8. Immunohistochemistry

Paraffin-embedded tissue sections were processed using standard immunohistochemical protocols. Endogenous peroxidase activity was quenched, and nonspecific binding was blocked prior to incubation with primary antibodies. Antigen–antibody complexes were visualized using a signal amplification detection system (K150011, DakoCytomation, Kyoto, Japan).

### 2.9. Histopathological and Immunohistochemical Analysis

Tumor tissue samples were fixed in 10% buffered formalin and processed for paraffin embedding. Serial sections with a thickness of 4 μm were prepared for subsequent analyses. Sections were subjected either to hematoxylin and eosin staining for routine histopathological evaluation or to immunohistochemical staining to characterize immune cell components within the tumor microenvironment [26]. All histopathological and immunohistochemical assessments were independently performed by two investigators who were blinded to the experimental conditions.

### 2.10. Measurement of Macrophages

Macrophages and myofibroblasts in tumors were evaluated semi-quantitatively [26]. Briefly, F4/80^+^ macrophages were quantified in 10 high-power fields (×1000) per section. Fields were randomly selected in a systematic and unbiased manner across the tumor area, avoiding preferential selection of regions with the highest cell density. All evaluations were performed by an examiner blinded to the experimental groups to minimize bias.

### 2.11. Double-Color Immunofluorescence Analysis

Double-color immunofluorescence was performed to determine CX3CR1 localization. Deparaffinized sections were incubated with PBS containing 1% normal donkey serum and 1% BSA to reduce nonspecific reactions. Thereafter, the sections were further incubated with a combination of anti-F4/80 and anti-CX3CR1, anti-F4/80, anti-MMP-2, or anti-F4/80 and anti-TGF-β, as previously described [26].

### 2.12. Real-Time RT-PCR Analysis

Total RNA was isolated from whole tumor tissues or cultured ID8 cells using ISOGEN reagent (Nippon Gene, Tokyo, Japan) following the supplier’s protocol. Complementary DNA was generated from 3 μg of total RNA using Oligo(dT)15 primers and PrimeScript Reverse Transcriptase (Takara Bio, Shiga, Japan). Quantitative PCR analysis was conducted with SYBR Premix Ex Taq II and gene-specific primers (Takara Bio). PCR amplification and signal detection were performed on a Thermal Cycler Dice Real-Time System (TP800, Takara Bio) in accordance with the manufacturer’s guidelines. To allow for normalization across samples, expression levels of the target genes were normalized to those of *Actb*, which was analyzed in parallel for each sample.

### 2.13. Flow Cytometric Analysis of ID8 Cells

ID8 cell suspensions were incubated with 25 μg/ml Fc block (BD Biosciences, Piscataway, NJ, USA) for 15 min on ice to prevent nonspecific binding. ID8 cells were stained with anti-CX3CR1 antibody. Analysis was performed on a FACScan flow cytometer (BD Biosciences) using the FlowJo software (Ver. 11, Tommy Digital Biology, Tokyo, Japan).

### 2.14. Flow Cytometric Analysis of Peritoneal Macrophages Under the Physiological Conditions

Peritoneal macrophages under the physiological conditions were evaluated from WT or *Cx3cr1*^−/−^ mice. After the sacrifice, mice were subjected to peritoneal lavages with 10 mL of sterile saline containing 5 mM EDTA. Subsequently, RBC were lysed in ammonium chloride buffer (150 mM NH_4_Cl, 10 mM NaHCO_3_, 1 mM EDTA-tetrasodium salt), and the remaining cells were examined by FACS analysis using PE-conjugated anti-F4/80 mAb (clone BM8, 12-4801-82, Thermo Fisher Scientific). Data from normal WT and *Cx3cr1*^−/−^ mice were obtained under a separate animal protocol approved by the Animal Care and Use Committee of Wakayama Medical University (No. 1227, approved on 20 August 2024).

### 2.15. Statistics and Reproducibility

Data are presented as mean values with the corresponding standard error of the mean (SEM) for all evaluated parameters. Statistical analyses were conducted using Statcel3 software (OMS, Saitama, Japan) with oversight by a medical statistician. Comparisons between two groups in in vivo and in vitro experiments were performed using the Mann–Whitney *U* test. Survival outcomes were analyzed using Kaplan–Meier survival curves, and differences between groups were evaluated with the log-rank test. A *p* value of less than 0.05 was considered to indicate statistical significance.

## 3. Results

### 3.1. Detection of CX3CL1 and CX3CR1 in Healthy and Malignant Ovarian Tissue Samples

**Figure 1:**
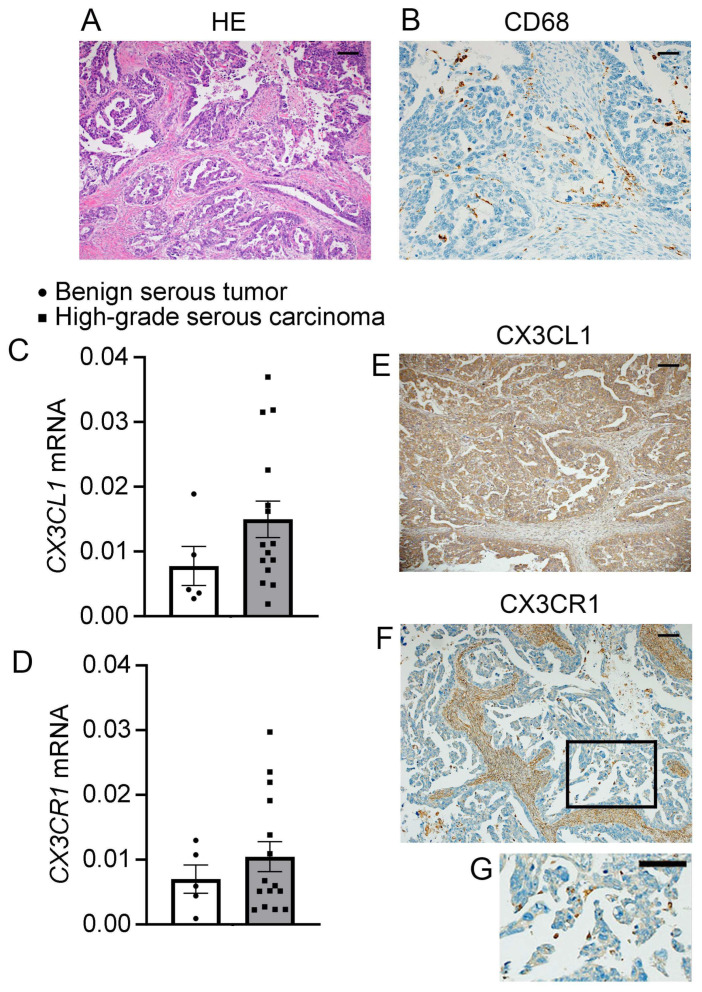
CX3CL1 and CX3CR1 expression in human ovarian carcinoma. (**A**) Histological findings of human epithelial ovarian cancer (EOC) samples with hematoxylin and eosin staining. Scale bar, 50 μm. (**B**) Immunohistochemical analysis was performed with anti-CD68 in human EOC samples. Scale bar, 50 μm. (**C**,**D**) *CX3CL1* (**C**) and *CX3CR1* (**D**) expression in human ovarian tumor samples. *CX3CL1* and *CX3CR1* expression were examined by real-time polymerase chain reaction. All values represent mean ± standard error of the mean (n = 4). vs. benign serous tumor. (**E**,**F**) Immunohistochemical analyses of CX3CL1 (**E**) and CX3CR1 (**F**) in human high-grade serous carcinoma. Scale bars, 50 μm; original magnification, ×200. (**G**) A highly expanding organizational structure within the framework of. Scale bar, 50 μm.

Figure 1A shows the histological findings of human EOC samples (HE staining). Immunohistochemically, infiltrated CD68^+^ macrophages were observed in EOC lesions (Figure 1B). mRNAs for *CX3CL1* and *CX3CR1* were detected by RT-PCR in five healthy human ovarian and 15 human EOC samples (Figure 1C,D). Although there were no significant differences in the expression of both genes between EOC samples and normal ovarian samples, EOC samples showed relatively but not significantly higher *CX3CL1* expression than normal ovarian tissues (Figure 1C,D). As shown in Table 1, the histological subtypes were heterogeneous, such as serous, mucinous, endometrioid, and clear cell adenocarcinoma. Thus, we compared the expression of *CX3CL1* and *CX3CR1* between histologically identical high-grade serous carcinoma and benign serous tumor. Benign superimposed tumors and serous adenocarcinoma tissues were histopathologically diagnosed by one of the authors (Y. H. T.), who specializes in ovarian cancer histopathology. The expression of both genes was significantly higher in the group of high-grade serous carcinoma than in that of benign serous tumor (Figure 1E,F). Additionally, immunohistochemical analysis showed that CX3CL1 and CX3CR1 proteins were highly expressed in ovarian tissue samples (Figure 1G). Immunohistochemical analysis revealed distinct distribution patterns of CX3CL1 and CX3CR1 in human ovarian cancer tissues. CX3CL1 staining was predominantly observed in tumor epithelial cells. In contrast, CX3CR1 staining was mainly detected in stromal regions rather than being uniformly expressed in tumor cells. Actually, some of the infiltrating immune cells within the tumor microenvironment expressed CX3CR1. Collectively, these observations implied that the CX3CL1–CX3CR1 axis might be involved in the progression of ovarian cancer.

### 3.2. CX3CL1 Enhanced the Migration of ID8 Cells, but Not Their Proliferation

**Figure 2:**
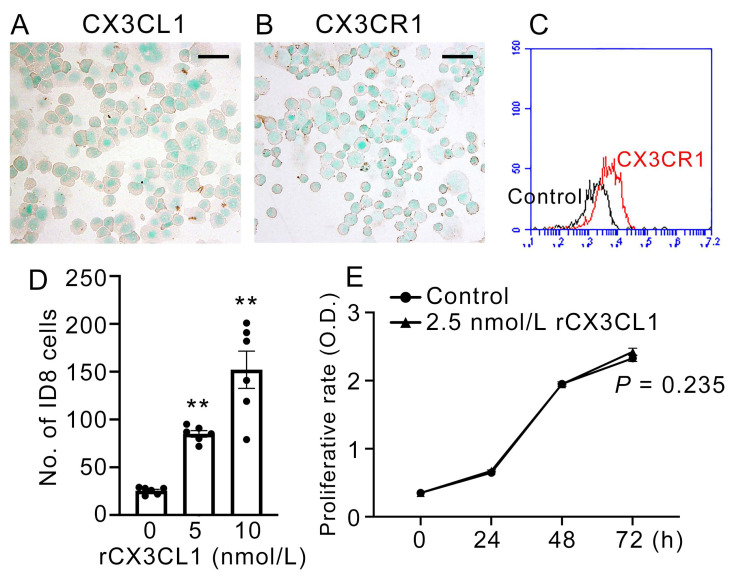
ID8 cells expressed both CX3CL1 and CX3CR1. (**A**,**B**) Immunohistochemical analyses of CX3CL1 (**A**) and CX3CR1 (**B**) expression in ID8 cells. Scale bar, 20 μm. (**C**) Flowcytometric analysis of CX3CR1 expression in ID8 cells. The black curve represents the isotype control used as a negative control for CX3CR1 staining. The red curve indicates CX3CR1 expression. Axes are labeled as fluorescence intensity (*x*-axis) and cell count (*y*-axis). (**D**) Cell migration assay of ID8 cells with recombinant CX3CL1 (rCX3CL1). All values represent mean ± standard error of the mean (SEM) (six independent experiments). ** *p* < 0.01 vs. without rCX3CL1. (**E**) Cell proliferative assay of ID8 cells with rCX3CL1. All the values represent mean ± SEM.

Immunohistochemical and flow cytometric analyses showed that ID8 cells, a murine ovarian cancer cell line, expressed CX3CL1 and CX3CR1 (Figure 2A–C). When the ID8 cells were treated with CX3CL1 in the next series, cell migration significantly increased in a dose-dependent manner (Figure 2D); however, cell proliferation rate was not enhanced (Figure 2E). These observations imply that the CX3CL1–CX3CR1 axis may be involved in ovarian cancer progression.

### 3.3. CX3CR1 Deletion Inhibited ID8-Inocluted Ovarian Tumor Growth and Progression

**Figure 3:**
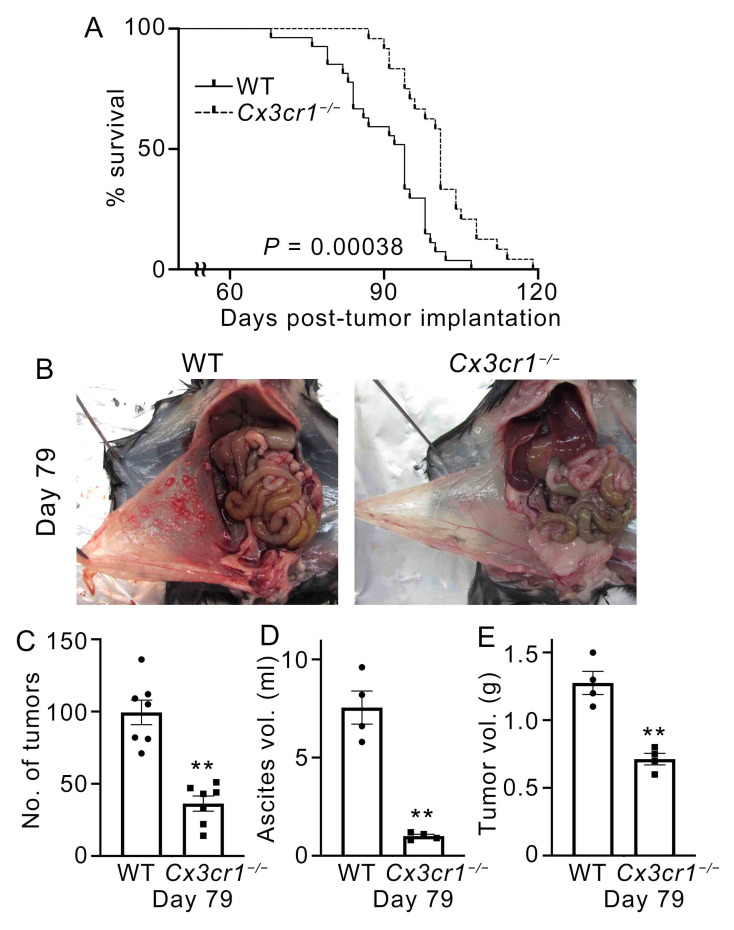
ID8-inoculated ovarian cancer was suppressed in *Cx3cr1* deficiency. (**A**) Survival ratio of ID8 cells challenged wild-type (WT) (n = 24) and *Cx3cr1^−/−^* (n = 27) mice. (**B**) Macroscopic evaluation of intraperitoneal tumor numbers in WT and *Cx3cr1^−/−^* mice on day 79. (**C**) The number of intraperitoneal tumors in WT and *Cx3cr1^−/−^* mice on day 79 (n = 7). (**D**) Ascites in WT and *Cx3cr1^−/−^* mice were measured on day 79 (n = 4). (**E**) Tumor volumes in the peritoneal cavity of WT and *Cx3cr1^−/−^* mice were measured on day 79 (n = 4). All values represent mean ± standard error of the mean. ** *p* < 0.01 vs. WT mice.

We examined the pathophysiological roles of the CX3CL1–CX3CR1 axis in ovarian cancer progression in vivo using a mouse syngeneic model of peritoneal dissemination. ID8 cells (5 × 10^6^ cells/mouse) were inoculated intraperitoneally into WT and *Cx3cr1^−/−^* mice. The *Cx3cr1^−/−^* mice exhibited significantly longer survival times (Figure 3A). Consistent with this result, the number of intraperitoneal tumors in the *Cx3cr1^−/−^* mice was lower than that in the WT mice on day 79 after ID8 inoculation (Figure 3B,C). On day 79, both ascites and tumor volumes in the peritoneal cavity from the *Cx3cr1^−/−^* mice were significantly reduced compared with those in WT mice (Figure 3D,E). In the ID8 intraperitoneal model, tumor nodules were predominantly distributed on peritoneal surfaces, including the mesentery and peritoneal lining. Although regional variability in tumor distribution was observed, overall tumor burden—assessed by the number and size of disseminated nodules within the peritoneal cavity—was markedly reduced in *Cx3cr1*-deficient mice compared with wild-type (WT) mice. Based on these results, the absence of CX3CR1 in host cells improved survival after tumor implantation.

### 3.4. Accumulation of CX3CR1^+^ Macrophages in the Abdominal Cavity of WT Mice

**Figure 4:**
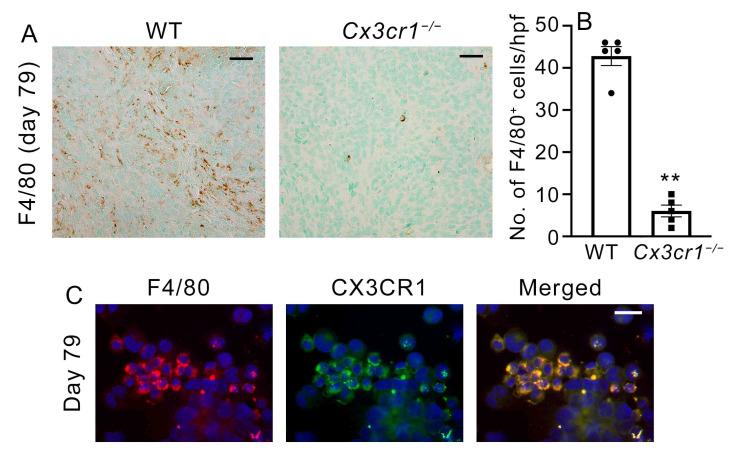
Tumor macrophages express CX3CR1. (**A**) Immunohistochemical analysis of F4/80^+^ macrophages in tumor tissues from wild-type (WT) and *Cx3cr1^−/−^* mice (day 79). Representative results from six independent experiments are shown (scale bar, 50 μm). (**B**) The number of F4/80^+^ macrophages in tumor tissues of WT and *Cx3cr1^−/−^* mice (day 79). Values represent mean ± standard error of the mean (n = 5). ** *p* < 0.01 vs. WT mice. (**C**) A double-color immunofluorescence analysis with anti-F4/80 and anti-CX3CR1 in ascites cells of WT mice (scale bar, 20 μm).

Immunohistochemical analysis demonstrated marked infiltration of F4/80^+^ macrophages in tumor tissues of WT mice (Figure 4A,B). Furthermore, double-color immunofluorescence analysis revealed that CX3CR1 was co-localized with F4/80^+^ macrophages (Figure 4C), indicating that tumor-associated macrophages represent a major CX3CR1-expressing population within the tumor microenvironment. These findings support that CX3CR1 expression in tumor tissues is largely attributable to infiltrating macrophages. On the other hand, flow cytometric analysis of unstimulated peritoneal exudate cells demonstrated that macrophage numbers were comparable between WT and *Cx3cr1*-deficient mice under physiological conditions, suggesting that the reduced macrophage accumulation in tumors of *Cx3cr1*-deficient mice was not due to a systemic deficiency of macrophages. Collectively, these observations suggest that CX3CR1-dependent macrophage recruitment would be essential for the progression of intraperitoneal ovarian cancer.

### 3.5. CX3CR1 Deficiency Reduced MMP-2 and TGF-β Expression

**Figure 5:**
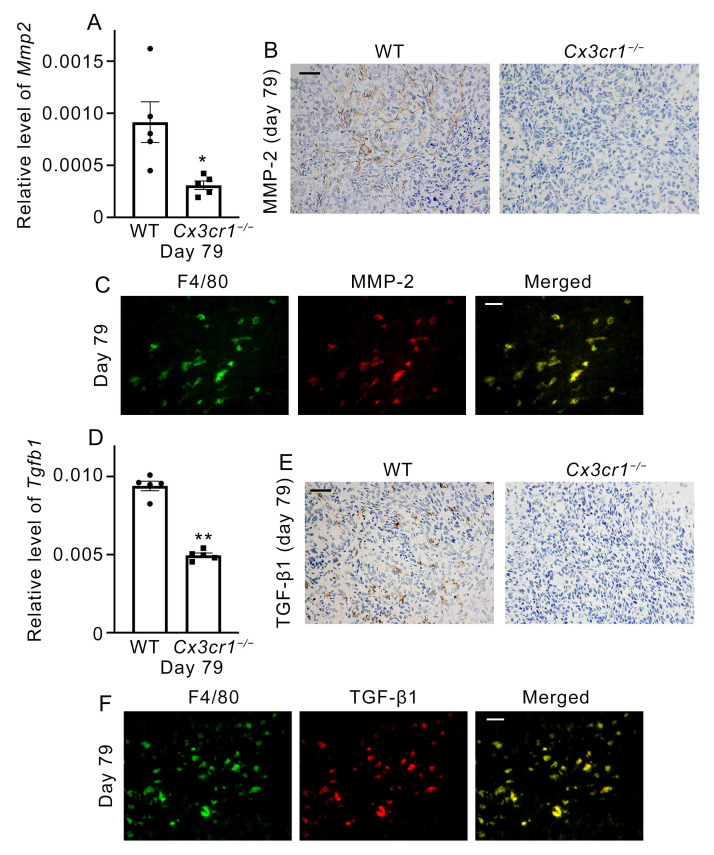
CX3CR1 deficiency suppressed MMP-2 and TGF-β in tumors. (**A**) *Mmp2* expression in tumor tissues of wild-type (WT) and *Cx3cr1^−/−^* mice (day 79). Values represent mean ± standard error of the mean (SEM) (n = 5). * *p* < 0.05, vs. WT mice. (**B**) Immunohistochemical analysis of matrix metalloprotease-2 (MMP-2) protein in tumor tissues of WT and *Cx3cr1^−/−^* mice (day 79). Scale bar, 50 μm. (**C**) Double-color immunofluorescence analysis with anti-F4/80 and anti-MMP-2 in tumor tissues of WT mice (day 79; scale bar, 20 μm). (**D**) *Tgfb1* expression in tumor tissues of WT and *Cx3cr1^−/−^* mice (day 79). Values represent mean ± SEM (n = 5). ** *p* < 0.01 vs. WT mice. (**E**) Immunohistochemical analysis of TGF-β1 protein in tumor tissues of WT and *Cx3cr1^−/−^* mice (day 79). Scale bar, 50 μm. (**F**) A double-color immunofluorescence analysis with anti-F4/80 and anti-TGF-β1 in tumor tissues of WT mice (day 79; scale bar, 20 μm).

*MMP2* expression in tumor samples was significantly reduced in *Cx3cr1^−/−^* mice compared with that in WT mice (Figure 5A). Consistent with this result, immunohistochemical analysis demonstrated that MMP-2 expression was attenuated in *Cx3cr1^−/−^* mice compared with that in WT mice (Figure 5B). Moreover, double-color immunofluorescence analysis demonstrated that F4/80^+^ macrophages in tumor tissues were the source of MMP-2 (Figure 5C). Additionally, *TGF-β* expression in murine tumor samples was significantly reduced in *Cx3cr1^−/−^* mice compared with that in WT mice (Figure 5D). Consistent with this result, immunohistochemical analysis demonstrated that TGF-β protein was attenuated in *Cx3cr1^−/−^* mice compared with that in WT mice (Figure 5E). Infiltrated F4/80^+^ macrophages in the tumor tissue could be a source of TGF-β (Figure 5F). In the double immunofluorescence analysis, the observed overlap between F4/80 and MMP-2 or TGF-β signals should be interpreted with caution. Given that F4/80 is a membrane-associated marker and MMP-2 and TGF-β are secreted proteins that may transiently localize intracellularly, complete subcellular colocalization is not necessarily expected. In addition, the absence of nuclear staining limits precise assessment of cellular localization. Therefore, the present data should be interpreted as indicating that MMP-2 and TGF-β signals are predominantly associated with F4/80^+^ macrophage populations at the cellular level, rather than demonstrating strict subcellular colocalization. However, these observations indicated that the CX3CL1–CX3CR1 axis is involved in MMP-2 activation and TGF-β production in the TME, eventually leading to tumor progression.

## 4. Discussion

This study demonstrated that the CX3CL1–CX3CR1 axis drives intraperitoneal progression of ovarian cancer through the accumulation of CX3CR1^+^ macrophages that release MMP-2 and TGF-β to the TME. Upregulation of CX3CL1–CX3CR1 with macrophage infiltration in human EOC tissues, enhanced migration of ID8 cells upon CX3CL1 stimulation, and improved survival with reduced tumor burden in *Cx3cr1*-deficient mice all support the tumor-promoting role of this pathway [10,20,22,27].

Specifically, *Cx3cr1* deficiency markedly reduced the infiltration of F4/80^+^ macrophages co-expressing MMP-2 and TGF-β. Double staining confirmed their colocalization, indicating that CX3CR1-dependent macrophages were the major source of these tumor-promoting factors. However, this study provides a new perspective by identifying a CX3CR1-dependent macrophage subset defined by its functional production of MMP-2 and TGF-β, rather than relying on the conventional M1/M2 polarization framework [13,14,15,23,24]. Macrophage-derived MMP-2 and TGF-β likely contribute to tumor progression through distinct but complementary mechanisms. MMP-2 plays a central role in extracellular matrix degradation, thereby facilitating tumor cell invasion and dissemination within the peritoneal cavity. In contrast, TGF-β exerts pleiotropic effects, including enhancement of tumor cell invasiveness, activation of cancer-associated fibroblasts, and suppression of antitumor immune responses. Thus, the coordinated production of MMP-2 and TGF-β by CX3CR1-dependent macrophages may promote both structural remodeling of the tumor microenvironment and establishment of an immunosuppressive niche that supports tumor progression.

MMP-2 and TGF-β are key mediators of ovarian cancer invasion and metastasis. TGF-β enhances tumor cell invasiveness by inducing MMP activity and activating cancer-associated fibroblasts [20,28]. MMP-2 promotes early peritoneal adhesion and invasion, and is expressed by both tumor epithelium and stromal cells. MMP-2 was selected as a representative effector molecule because of its established role in extracellular matrix remodeling and peritoneal dissemination in ovarian cancer. Our observations demonstrate that CX3CR1^+^ macrophages are the major source of MMP-2. Although a direct regulatory link between the CX3CL1–CX3CR1 axis and MMP-2 expression has not been fully established, our findings suggest that this axis indirectly influences MMP-2 levels by promoting the recruitment of macrophages that serve as a major source of this protease within the tumor microenvironment [23,24]. Thus, the reduction in MMP-2/TGF-β^+^ macrophages in *Cx3cr1*-deficient mice is mechanistically consistent with the attenuated peritoneal dissemination and improved outcomes.

In addition, it should be noted that other cell types, including tumor cells and stromal fibroblasts, may also contribute to MMP-2 and TGF-β production. Therefore, residual mRNA expression observed in *Cx3cr1*-deficient tumors may, at least in part, originate from these non-macrophage cell populations. Given that MMP-2 and TGF-β are produced by multiple cell types, direct targeting of these molecules may have limited specificity. In this context, targeting upstream pathways such as the CX3CL1–CX3CR1 axis may provide a more selective strategy by reducing the recruitment of macrophages that serve as major sources of these mediators.

CX3CR1 expression in ovarian cancer and bone marrow-derived cells further underscores the dual role of this axis. Previous studies have demonstrated that CX3CR1 contributes to motility and peritoneal implantation of EOC cells [10,18] and that CX3CR1^+^ myeloid populations are functionally relevant in ascites and tumor tissues [22,27]. Moreover, CX3CL1–CX3CR1 signaling regulates TAM accumulation during skin carcinogenesis, and CX3CR1 blockade suppresses tumor growth [20]. Similar mechanisms have been reported for breast cancer and glioma [21,28,29]. The similarities between ovarian and non-ovarian malignancies reinforce the broad oncological relevance of this chemokine axis.

The classical M1/M2 paradigm classifies macrophages into pro-inflammatory (M1) and anti-inflammatory (M2) subsets [13,14]. Our previous observations [20,30] demonstrated that CX3CR1 was predominantly expressed on M2 macrophages, and that CX3CR1^+^ M2 was essentially involved in skin carcinogenesis. However, accumulating evidence indicates that macrophages exhibit considerable plasticity and exist along a continuum of activation states influenced by the local microenvironment [15,31]. In this context, classification based solely on M1/M2 markers may be overly simplistic. Therefore, in the present study, we focused on functional outputs, identifying a CX3CR1-dependent macrophage subset characterized by MMP-2 and TGF-β production, rather than assigning macrophages to fixed polarization states. This functional characterization may prove to be more informative than broad phenotypic classification, suggesting that future therapeutic interventions should target effector programs rather than static polarization states. Single-cell and spatial transcriptomics technologies can delineate CX3CR1-dependent subsets in greater detail, providing molecular signatures to guide precision immunotherapy [31].

Our results support the clinical use of CX3CR1 as a potential therapeutic target. In this context, TAM-derived MMP-2 and TGF-β may represent a therapeutic vulnerability downstream of the CX3CL1–CX3CR1 axis. Small-molecule inhibitors, such as AZD8797 and KAND567, as well as mAbs against CX3CR1, are currently under development, with preclinical efficacy in cardiovascular and oncological contexts [32,33]. The dual benefits of inhibiting CX3CR1—directly impairing tumor cell motility and reducing infiltration of macrophages producing MMP-2 and TGF-β—highlight its translational potential. Furthermore, TAM-derived TGF-β has been implicated in ascites formation and metastatic progression [24,28], suggesting that CX3CR1 blockade could alleviate both tumor burden and associated morbidity. Although CX3CR1^+^ immune cells have been implicated in antitumor responses in other malignancies, including lung cancer [34], the relevance of these findings to ovarian cancer remains unclear and warrants further investigation.

Despite these promising findings, some study limitations must be acknowledged. The relatively small number of human samples analyzed in this study limits the generalizability of our conclusions. Additionally, although colocalization of MMP-2 and TGF-β in F4/80^+^ macrophages was confirmed, more comprehensive phenotyping is needed to define the full repertoire of CX3CR1-dependent TAM subsets. Future studies should integrate multi-omics approaches, including single-cell RNA sequencing and proteomics, to provide a comprehensive map of the TAM landscape in ovarian cancer. Furthermore, functional validation of the CX3CR1 blockade in humanized models and clinical samples is critical to confirm its translational potential. Furthermore, because this study employed a global *Cx3cr1* knockout model, we cannot completely exclude the potential contribution of CX3CR1 deficiency in non-macrophage cell populations, and further studies using cell-type-specific approaches are warranted. Because bulk RNA analysis of ovarian tissue includes heterogeneous cell populations and most ovarian cancers originate from the epithelial layer, comparisons between normal ovarian tissue and ovarian cancer should be interpreted with caution.

Collectively, our findings highlight the dual role of the CX3CL1–CX3CR1 axis in ovarian cancer progression, acting both on tumor cells and within the tumor microenvironment. CX3CR1 expression in tumor cells contributes to migratory and adhesive properties, whereas CX3CR1-dependent recruitment of macrophages promotes the accumulation of cells that produce MMP-2 and TGF-β, thereby facilitating tumor growth, invasion, and peritoneal dissemination. This integrated view underscores the importance of CX3CR1 as a regulator of both cancer cell–intrinsic behavior and the surrounding stromal compartment. Although multiple chemokine pathways, including CXCR3- and CCR-dependent axes, have been implicated in macrophage recruitment, our findings suggest that the CX3CL1–CX3CR1 axis plays a dominant role in this model. The unique ability of CX3CL1 to function in both membrane-bound and soluble forms may facilitate efficient macrophage adhesion and retention within the tumor microenvironment. Nevertheless, potential contributions of other chemokine pathways cannot be excluded, and incomplete compensation in the absence of CX3CR1 may reflect functional specialization among these signaling axes. In addition, although this study focused on macrophages, the CX3CL1–CX3CR1 axis may also influence the recruitment of other immune cell populations, including T cells and NK cells. Because we did not comprehensively analyze these subsets, their potential contribution to the observed phenotypes cannot be excluded. Future studies are required to clarify the broader immunological impact of this chemokine axis within the tumor microenvironment.

## 5. Conclusions

From a therapeutic perspective, targeting the CX3CL1–CX3CR1 axis may provide dual antitumor effects by simultaneously impairing tumor cell motility and limiting the recruitment of pro-tumorigenic macrophages. However, the relatively small number of clinical samples analyzed in this study represents a limitation. Inclusion of publicly available datasets, such as The Cancer Genome Atlas, would provide external validation and increase statistical robustness; therefore, such analyses will be incorporated in future studies to further validate and extend our findings.

## Figures and Tables

**Table 1:** Summary of ovarian cancer samples.

Variable		No. of Patients (%)
Age at surgery, median (range)		50 years (24–71 years)
Histological subtype		
	High-grade serous carcinoma	4 (26.7)
	Endometrioid carcinoma	5 (33.3)
	Mucinous carcinoma	4 (26.7)
	Clear cell carcinoma	2 (13.3)
FIGO Stage		
	I	6 (40.0)
	II	2 (13.3)
	III	5 (33.3)
	IV	2 (13.3)
Neoadjuvant chemotherapy		
	Yes	1 (6.7)
	No	14 (93.3)
Type of surgery		
	Primary debulking surgery	11 (73.3)
	Interval debulking surgery	3 (20.0)
	Exploratory laparotomy (diagnostic)	1 (6.7)
Residual disease after surgery		
	No gross residual disease (R0)	12 (80.0)
	Gross residual disease	3 (20.0)
Recurrence status		
	Recurred	6 (40.0)
	No recurrence	9 (60.0)
Vital status at 10 years		
	Alive	10 (66.7)
	Dead	4 (26.7
	Lost to follow-up	1 (6.7)

## Data Availability

The datasets used and/or analyzed in the current study are available from the corresponding author upon reasonable request.

## Abbreviations

The following abbreviations are used in this manuscript:

CX3CL1	Chemokine (CX3C motif) ligand 1
CX3CR1	CX3C motif chemokine receptor 1
MMP	Matrix metalloprotease
TAM	Tumor-associated macrophage
TGF-β	Transforming growth factor-β
VEGF	Vascular endothelial growth factor
ECM	Extracellular matrix
mAbs	Monoclonal antibodies
RT-PCR	Reverse transcriptase-polymerase chain reaction
WT	Wild-type
PBS	Phosphate-buffered saline
BSA	Bovine serum albumin
TME	Tumor microenvironment
